# Multiple entanglements of different cell death pathways, in which caspase-8 and BID interact with cardiolipin*, have been identified

**DOI:** 10.3389/fcell.2025.1667611

**Published:** 2025-11-11

**Authors:** Patrice X. Petit

**Affiliations:** National Center for Scientific Research, CNRS UMR 8003, Paris City University, Saint-Pères Paris Institute for Neuroscience (SSPIN), Team “Mitochondria, Apoptosis and Autophagy Signaling”, Paris, France

**Keywords:** BID, cardiolipin, cell death, cardiolipin peroxidation, giant unilamellar vesicles, mitochondria, outer mitochondrial membrane, tBID

## Abstract

Mitochondria play a central role in cellular bioenergetics, being major counterparts in ATP production and thus in the maintenance of cells, but they are also key mediators of different types of cell death (apoptosis, necroptosis, ferroptosis, etc.) and are among the main players in autophagy. With respect to death receptor-mediated apoptosis, activation of the mitochondrial pathway is required for the induction of apoptosis in cells (extrinsic pathway), referred to as “type II” cells. In type I cells, activation of the extrinsic pathway through a large amount of caspase-8 allows direct activation of caspase-3 and is sufficient to induce apoptosis. This small review is dedicated to the often forgoten molecule of the BCL-2 family, BID. Special emphasis will be placed on the importance of the cardiolipin/caspase-8/BID platform located at the outer mitochondrial membrane surface that generates tBID, which is the actor of BAX/BAK delocalization and oligomerization at the mitochondrial surface and then transmits death signals in the apoptotic pathway. New insights into the regulation of caspase-8 and BID have emerged, and their originality in the context of their activation and function will be highlighted. We will focus on results from biophysical studies of artificial membranes, i.e., lipid-supported monolayers or giant unilamellar vesicles containing cardiolipin. The destabilization of mitochondrial bioenergetics by tBID insertion at the mitochondrial contact site is presented. Since it inhibits the electron transfer chain, superoxide anion generation is essential for BAX oligomerization. We will take you on a journey through these new developments that reveal a surprisingly high degree of redundancy and crosstalk between the apoptotic, necroptotic, and pyroptotic cell death pathways. Taken together, the mitochondrial contact site and cristae organization system (MICOS) is a critical determinant of mitochondrial membrane architecture and physiology. Its close crosstalk with many other mitochondrial protein machineries identifies the MICOS as a central hub in an interwoven network that ensures mitochondrial functionality and integration into the cellular context. It is becoming increasingly clear that the activation platform built around caspase-8/cardiolipin and BID is involved in multiple types of cell death, including apoptosis, ferroptosis (oxytosis), necroptosis and autophagic death.

## Graphical Abstract

**Figure:**
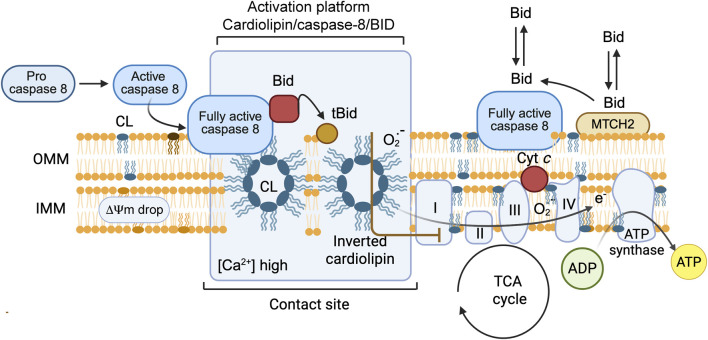

## 1 Introduction

Apoptosis, also known as programmed cell death, plays an important role in tissue development and maintenance (Danial and Korsmeyer, 2004; Kroemer et al., 1995). The proteins of the BCL-2 (B-cell lymphoma 2) family, which form an extensive group of apoptosis regulators, coordinate to modulate the destabilization and permeabilization of the mitochondrial outer membrane (MOM) and promote the release of numerous proteins from the intermembrane space (Wang, 2001; Youle and Strasser, 2008; van Loo et al., 2002), which allows programmed cell death to occur. Mitochondria are double-membraned subcellular organelles found in the cells of all multicellular eukaryotes. This organelle is widely regarded as the “powerhouse” of cellular metabolism, catalyzing the production of adenosine triphosphate (ATP) via oxidative phosphorylation (OXPHOS) (McBride et al., 2006; Lane and Martin, 2010).

**FIGURE 1:**
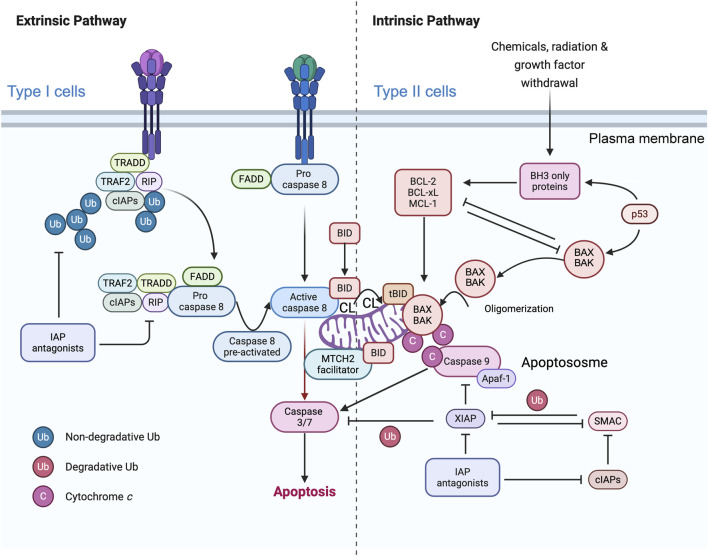
Simplified representation of the role of mitochondria in caspase-8 signaling in the context of type I and type II cells. The binding of CD95 (or TRAIL) to their respective receptors leads to receptor trimerization and the formation of the death-inducing signaling complex (DISC). The adaptor protein FADD is recruited to the DISC, where the death domains (DDs) of both proteins interact. Procaspase-8 is subsequently recruited to the protein complex, where it interacts with FADD via the death effector domains (DEDs). cFLIP can compete with caspase-8 for binding to FADD. Therefore, high levels of cFLIP can prevent caspase-8 activation in the DISC. DISC-activated caspase-8 initiates a caspase cascade by cleaving caspase-3. In type I cells, activation of the extrinsic pathway is sufficient to induce TRAIL- and CD95-induced apoptosis, whereas in type II cells, BID protein cleavage is required for TRAIL- and CD95L-induced apoptosis. Caspase-8 activated at the MOM (more precisely, at the contact sites or within the MICOS domain) cleaves BID to tBID, which is inserted into the membrane where it disrupts mitochondrial bioenergetics and allows BAX/BAK to delocalize to the MOM and initiate the mitochondrial apoptosis pathway, leading to the release of cytochrome *c* (Cytochrome c*)* and Smac/DIABLO from the mitochondria. MTCH2/MMP may facilitate the translocation of tBID to the outer mitochondrial membrane (MOM), but the main hypothesis involves the integration of caspase-8 into cardiolipin (CL)-rich domains of the MOM, resulting in full activation of this caspase, which can then directly access and cleave its substrate BID. After release from the mitochondria, Cytochrome *c,* together with Apaf-1 and dATP, forms the apoptosome, an activation platform for caspase-9. Smac/DIABLO counteracts the inhibitory function of XIAP, allowing full activation of caspase-3 and -9 and ultimately leading to cell death. This schematic interpretation was drawn partly with the Scientific Image and Illustration Software/Biorender and is inspired by Scaffidi et al. (1998) but remains under the copyrigth of @Patrice X. Petit.

The semipermeable outer membrane controls the exchange of material and information with other cellular compartments. The inner membrane, with its extended, highly folded surface, is the site of selective transport and energy coupling reactions. It can be considered laterally heterogeneous, as it has spatial subdivisions that are inner limiting membranes and tubular or lamellar cristae membranes, which house the units of the electron transport chain. The MOM, inner limiting membrane and cristae meet at cristae junctions where the mitochondria contact sites (Brdiczka et al., 2006), which is called the “cristae organizing system (MICOS)” (Eramo et al., 2020; Benaroya, 2024), act as a membrane that forms and connects scaffolds (Dietz et al., 2021). A special architecture is essential for the multiple functions of the mitochondrion (Benaroya, 2024). The outer and inner mitochondrial membranes are composed of a mosaic of proteins and phospholipids, with a distinct intermembrane space between them. The inner mitochondrial membrane undergoes invagination and curves inward into the mitochondrial matrix, forming cristae (Dietz et al., 2021). Numerous assembled complexes involved in OXPHOS and ATP synthesis have been identified on cristae. The crista membrane, as part of the inner membrane, complicates the process of folding and maintaining mitochondrial integrity in response to stress. Upon binding, FasL induces Fas receptor multimerization and recruits the FADD/MORT1 adaptor and the pro-form of caspase-8 to the death domain of the receptor, leading to the formation of the death-inducing signaling complex (DISC) (Peter et al., 1999; Peter and Krammer, 2003) (Figure 1). Dimerization of caspase-8 at the DISC triggers its activation by a conformational change, followed by autoproteolytic processing and release into the cytosol. Preactivated caspase-8 then binds to cardiolipin for further activation. The subsequent apoptotic program can kill cells via two different pathways, type I or type II (Youle and Strasser, 2008; van Loo et al., 2002). Thus, cells with high levels of XIAP are unable to directly activate caspase-3 following DISC-induced caspase-8 activation, indicating that BID cleavage and subsequent mitochondrial outer membrane permeability (MOMP) are prerequisites for the onset of apoptosis in these cells. Therefore, the expression or absence of XIAP is an important factor in classifying cells as type I or type II with respect to death receptor-mediated apoptosis (Scaffidi et al., 1999; Scaffidi et al., 1998).

In type I cells (intrinsic cell death pathway), caspase-8 (in quite elevated amounts) directly processes and activates the effector caspases -3 and -7, which are sufficient to execute cell destruction (Scaffidi et al., 1999; Scaffidi et al., 1998). In type II cells (extrinsic cell death pathway), apoptosis induction also requires amplification (Scaffidi et al., 1999; Scaffidi et al., 1998) after caspase-8 to cleave the proapoptotic BH3-only BCL-2 family member BID, which is located in apposition to capase-8, forming tBID, which is primarily inserted into the MOM through cardiolipin and activates the proapoptotic BCL-2 family members BAX and BAK. The oligomerization of BAX/BAK induces MOMP, which results in the release of cytochrome *c* (Youle and Strasser, 2008) and other proteins in the mitochondrial intermembrane space (van Loo et al., 2002). Once in the cytosol, cytochrome *c* binds to Apaf-1 and recruits procaspase-9 into a complex called the “apoptosome,” which processes and activates the effector caspases -3 and -7 (Lane and Martin, 2010).

The reasons for the differences between type I and type II cells are not fully understood (Brdiczka et al., 2006), although differences in the expression levels of inhibitors of the death receptor signaling cascade, such as c-FLIP or XIAP (Figure 1), may provide an explanation. FLIP is an inhibitory caspase-8 analog that acts on the DISC (Eramo et al., 2020), whereas XIAP blocks the enzymatic activity of caspase-9, -3 and -7 (Lane and Martin, 2010). It is conceivable that the caspase-inhibitory effect of XIAP can be overcome by apoptogenic proteins such as Smac/DIABLO and Htr2A/Omi, which are released from mitochondria together with cytochrome *c* (van Loo et al., 2002). These proteins bind to XIAP, neutralizing its caspase-binding activity and/or targeting it for proteasomal degradation (Jost et al., 2009). Consequently, it has been hypothesized that cells with high levels of XIAP require tBID/mitochondria-mediated amplification of the caspase cascade to overcome XIAP-mediated inhibition of caspases (van Loo et al., 2002). The transfer of FADD/MORT1 and the pro-form of caspase-8 to the cytoplasmic death domain of the receptor has been observed, leading to the formation of the death-inducing signaling complex (DISC) (McBride et al., 2006). Consequently, cells with elevated levels of XIAP may require tBID/mitochondria-mediated amplification to overcome XIAP-mediated inhibition of caspases (McBride et al., 2006).

## 2 BID, a BH3-only member

### 2.1 BID, as a BH3-only member of the BCL-2 family of proteins

All members of the BCL-2 family have at least one of the four BCL-2 homology domains in common, and most share a transmembrane domain at the C-terminus (Willis and Adams, 2005; Willis et al., 2007; Kuwana et al., 2005; Kuwana and Newmeyer, 2003). BCL-2 s are divided into two groups with respect to their pro- or antiapoptotic functions. Within the proapoptotic subset (e.g., BID, BIM, and PUMA), which shares only the BH3 domain, these proteins have the ability to interact with BAX/BAK, promote their oligomerization at the MOM), and lead to their permeabilization (MOMP) (Bogner et al., 2010; Leber et al., 2007). This is the main model for the process involved in the initiation/activation of apoptosis. Another point to consider is that the antiapoptotic proteins of the BCL-2 family (e.g., BCL-2, BCL-XL, BCL-W and MCL-1) can bind to BAX or BAX (heterodimerization) to counteract their proapoptotic activity. A sensitizing role can be given to BH3-only proteins susceptible to binding antiapoptotic proteins and can induce BAX and BAK oligomerization, which ultimately results in MOMP (Willis and Adams, 2005; Willis et al., 2007; Leber et al., 2007).

The insertion of many BCL-2 family proteins into the mitochondrial membrane is an obligatory step, and their translocation from the cytosol to the mitochondrial membrane could be responsible for completely different interactions between members of the BCL-2 family (Lovell et al., 2008; Lutter et al., 2001; McDonnell et al., 1999).

**FIGURE 2:**
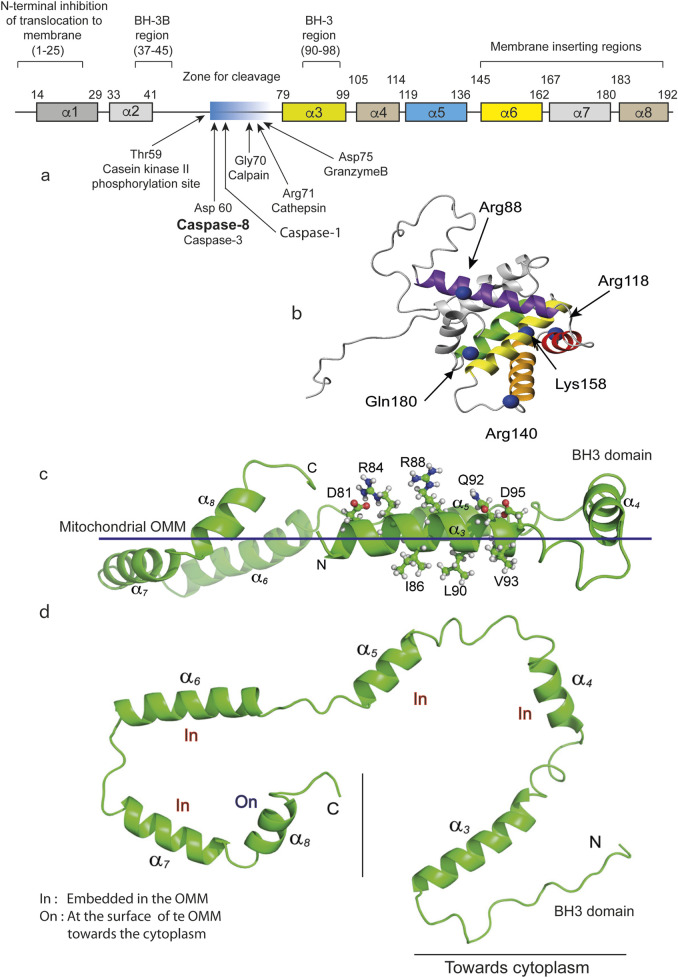
BID and tBID representations and their insertion into the mitochondrial contact sites. **(a)** Schematic linear representation of the entire BID molecule. Regions and residues of BID that regulate membrane binding and the proapoptotic function of the human BID protein. BID contains 195 amino acids and 8 a-helices. The numbers above each a-helix indicate the start and end amino acids for that helix. The regions that control the recruitment of proteins to membranes and their function, including the BH regions and domains thought to be inserted into membranes, are indicated above the proteins. The cleavage sites are indicated by arrows. Where appropriate, the amino acids corresponding to a particular region are indicated in parentheses. Individual amino acids that regulate membrane binding or that undergo posttranslational modifications that regulate membrane binding are indicated below the proteins, with the identity and number of each amino acid indicated in parentheses. **(b)** Positions to probe conformational changes and intermolecular contacts in BID. A ribbon representation of the BID structure (PDB ID: 2BID) shows the eight α-helices. The C-β carbons of the residues mutated to a cysteine for FRET assays are shown in blue, marked with an arrow and named directly on the image as cited by Gahl et al. (2016). **(c)** tBID adopts a C-shaped structure with both N- and C-termini in close conformations. C, tBID helices (aH3-aH8) are parallel to the putative membrane surface, with helices aH6 and aH7 being more embedded. For the membrane-associated tBID helix aH3, charged or polar residues (Asp81, Arg84, Arg88, Gln92, and Asp95) face away from the membrane surface and are exposed for potential interactions with other proteins. **(d)** On the opposite side of the helix α 3, residues with strong micelle interactions (Ile86, Ala87, Leu90, Val93, and Gly94) face the membrane surface. The flexible N-terminus (Gly61--Ser78) has been omitted for clarity. **(c,d)** Representations are directly inspired by Wang and Tjandra (2013).

BID, which simply stands for BH3-interacting domain death agonist, is a member of the BCL-2 family of proteins that act at the mitochondrial outer membrane surface (MOM) and whose interactions regulate membrane permeability, which has been shown to be a key event in apoptosis (Figure 2a). On the basis of all the current data and with the aim of validating the membrane association hypothesis, an “embedded together model” has been elaborated, with initial tBID insertion into the MOM and a subsequent final state of BAX/BAK activation, with MOM permeabilization (Willis et al., 2007) as the so-called point-of-no return (Kroemer et al., 1995). Great progress has been made in translating the structural information of the cytosolic BCL-2 family into the analysis of its membrane-associated forms (McDonnell et al., 1999) (Figures 2b,c).

This protein, BID, is the all-too-often forgotten BH-3-only protein. BID is a key feature in the crosstalk between extrinsic and intrinsic pathways, which is mediated by its cleavage by caspase-8 (Yin et al., 1999; Wang et al., 1996; Li et al., 1998), which is the main downstream mediator of the Fas or tumor necrosis α (TNFα) death receptor signaling pathways. Fas/CD95-induced apoptosis *in vivo* occurs through the so-called type II pathway, which requires the proapoptotic BH3-only BCL-2 family member BID for mitochondrial death signaling (Figure 1). Consequently, BID-deficient mice are protected from fatal hepatitis induced by anti-Fas antibody injection (Li et al., 1998).

The BH3-only protein BID, which binds to BCL-2 and BAX, was the rationale that led Wang et al. (1998) to clone BID for the first time. Further studies identified BID as a caspase-8 substrate (Gross et al., 1999) and led to the hypothesis that the C-terminal fragment of BID, called tBID (tcBID, p15 fragment), results from caspase-8 cleavage, accumulates at the mitochondrial surface and releases cytochrome *c* (Bogner et al., 2010; Lovell et al., 2008). The existence of a caspase-8 cleavage site at Asp60 of BID allows the formation of p15 (tcBID)- and p7 (tnBID)-linked fragments of BID (Kuwana et al., 2005; Lovell et al., 2008). However, the inhibition exerted by the N-terminal fragment (tnBID, p7) is immediately relieved by the proximity of the CL and the high affinity of tBID for the CL. The exposed hydrophobic residues on the p15 fragment increase in number and participate in the initiation of binding of the newly formed protein to the membrane (van Loo et al., 2002; Kuwana et al., 2005). The hydrophobic interactions between the two fragments were previously observed when recombinant caspase-8 was used to cleave BID, and octylglucoside was then used to separate the two fragments (Lovell et al., 2008) (Figure 2).

**FIGURE 3:**
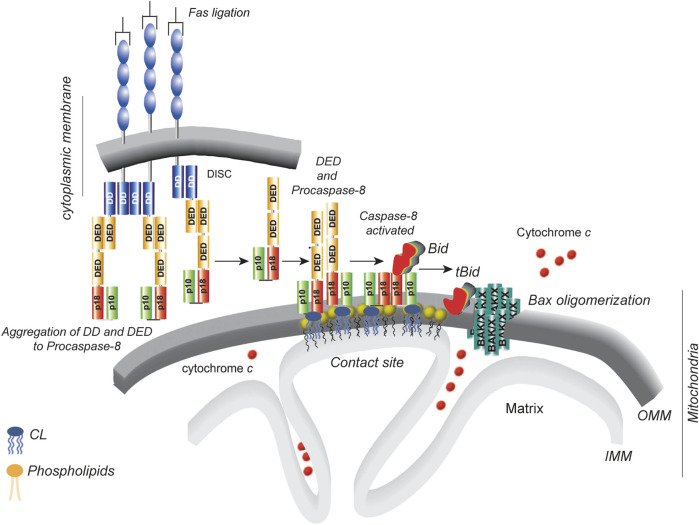
Localized production of active, cleaved tBID on cardiolipin platforms. The diagram illustrates the sequence of events that occur in type II cells (Wang et al., 1996). DD stands for the “death domain”, and DED stands for the “death effector domain.” The p10 and p18 domains constitute the catalytic core of caspase-8. The p43/p10 caspase-8 isoform consists of two DEDs, one p10 domain and one p18 domain. The cardiolipin-8 platform (yellow heads) at the contact sites between the inner and outer mitochondrial membranes produces active tBID as needed. This, in turn, causes BAK/BAX oligomerization and cytochrome *c* release. The diagram is taken from the comments of Scorrano (2008) on the article by Gonzalvez et al. (2008).

With respect to the cleavage of BID and the release of the p15 moiety, the presence of a proteo-lipidic membrane reminiscent of the MOM favored the spontaneous dissociation of p7 and p15 and the insertion of tBID into the membrane (Wang et al., 1996) (Figure 2). The authors of this proposal were indeed looking for a membrane target of BID. It is always surprising that fundamental issues that have not yet been solved are hidden in a corner of a princeps publication. In our case, a careful reading by Lutter et al. (2000) might have shortened the time to formulate the hypothesis that the CL is essential for caspase-8 and tBID (Figure 3). This very discrete information was indeed released from the initial work of Wang’s laboratory since the purified BID also contained a small amount of cardiolipin linked to it (Lutter et al., 2000).

In any case, tBID translocates to the MOM (Lutter et al., 2001; Gonzalvez et al., 2005a) and interacts with CL and its derivatives, including peroxidized CL (CLOOH) (Korytowski et al., 2011). The “simple” membrane insertion of tBID is essential for the efficient recruitment of cytosolic BAX to the MOM and further oligomerization (Lutter et al., 2001; Korytowski et al., 2011). BAX undergoes conformational changes during its interaction with the tBID-BH-3 domain, which allows membrane insertion and favors homo-oligomerization. This leads to pore formation in the mitochondrial outer membrane (MOM) and the release of multiple intermembrane space proteins, including cytochrome *c*, which activates the downstream executioner caspase-9 and -3 (Lovell et al., 2008; Lutter et al., 2000; Kriska et al., 2005; Raemy and Martinou, 2014). Moreover, tBID alone, without BAX and BAK, could act and provoke MOM permeabilization (deduced from experiments with artificial membranes) (Jalmar et al., 2010) along with the induction of membrane curvature changes that initiate mitochondrial bioenergetic destabilization (Jalmar et al., 2013).

The enhanced and accelerated apoptosis observed in CL-deficient cells (ΔCLS1 cells) is accompanied by a rapid decrease in membrane potential (ΔΨm) and increased cytochrome *c* (cytochrome c) release but not by decreased tBID binding (Choi et al., 2007). As the presence of cardiolipins is known to be vital for these functions, PGs, whose abundance is multiplied by five (x 5) in cardiolipin synthase-depleted cells, may substitute for cardiolipins for most of their essential functions under normal conditions, whereas their presence is essential under conditions of stress (Jalmar et al., 2010; Zaltsman et al., 2010). Alternatively, the mitochondrial carrier homolog 2 protein (MTCH2) may assume this role instead of missing the CL (Zaltsman et al., 2010). However, when both CL and MTCH2 were depleted (HCT116 cells), a significant reduction in tBID recruitment was observed, suggesting that CL and MTCH2 may have redundant functions in this process (Raemy et al., 2016), except that MTCH2 does not bind to caspase-8 or assume the final activation of caspase-8. Indeed, our view is that MTCH2 may facilitate the recruitment of BID/tBID to the MOM surface.

### 2.2 BID, caspase-8 and cardiolipin: a biophysical approach

Indeed, there is a consensus in death receptor-mediated apoptosis that the core of their signaling is triggered via a cardiolipin/caspase-8/BID (Kantari and Walczak, 2011; Scorrano, 2008) platform that targets tBID production and interaction with BAX/BAK (Raemy and Martinou, 2014). BAX oligomerization at the MOM induces membrane permeabilization and the release of proapoptotic factors, including cytochrome c, leading to caspase activation and cell disassembly. Importantly, BID can also be cleaved by several enzymes, not only caspase-8 but also calpain, cathepsin, and granzyme B (Figure 2a).

Importantly, the mitochondrial outer membrane is not a passive partner in this process, as membranes are required for both protein‒protein and protein‒lipid interactions. Simultaneous measurements of these interactions revealed an ordered series of steps required for (1) preactivated caspase-8 to acquire its full activation properties upon contact with cardiolipin, (2) BID to be recruited to activated caspase-8 (electrostatic interactions and docking), and (3) tBID to rapidly bind to membranes via interaction with the CL and when it is formed with monolysocardiolin (Figures 2c,d), where (4) tBID disrupts mitochondrial energetic functions and nearly interacts with delocalized BAX, causing (5) BAX insertion into membranes and (6) oligomerization, culminating in (7) membrane permeabilization (Figure 3). This view has been elaborated by many successive studies (Gonzalvez et al., 2005a; Gonzalvez et al., 2008; Gonzalvez and Gottlieb, 2007) and strongly argued in the concept of tBID and BAX “embedded together” (Leber et al., 2007; Lovell et al., 2008; Leber et al., 2010). In this context, BCL-XL prevents membrane-bound tBID from binding BAX. Bad releases tBID from BCL-XL, restoring both tBID binding to BAX and membrane permeabilization (Figure 3).

### 2.3 Proof of concept with DOPC/CL unilamellar giant vesicles

As a valuable premise of the further developed arguments concerning the relationship between tBID/BAX and CL, useful work has been established. Using confocal microscopy, fluorescence correlation spectroscopy and atomic force microscopy they showed that the behavior of the CL is tuned to promote the formation of membrane regions with double bilayers (apposed) or nonlamellar structures reminiscent of the “so-called” mitochondrial contact sites (Gonzalvez et al., 2005a; Gonzalvez et al., 2008; Gonzalvez and Gottlieb, 2007). In the presence of well-defined lipid bilayers, tBID and BAX are able to remodel membranes and stabilize curvature. Several lines of evidence have led to this working hypothesis. The team of Garcia-Saez has studied the effects of tBID and BAX using atomic force spectroscopy (Unsay et al., 2017). They used supported lipid structures mimicking the mitochondrial composition of two compositions: the simple one containing PC:CL at a ratio of 8:2 and the second one more prone to be similar to the mitochondrial outer membrane with the following composition, i.e., PC:PE:PI:PS:CL (with 48.5:27.2:9.9:10.0:4.4.4 mol%), which could be named “mitomix” (Horvath and Daum, 2013). The results are quite clear: tBID does not induce membrane permeabilization, but in its presence, the breakthrough force is much lower than usual. The effects of BAX depend on its oligomeric state. Monomeric BAX did not affect the properties of the membranes, whereas oligomerized BAX also lowered the breakthrough force of the membrane. In the context of pore formation, this implies a decrease in the line tension at the edge of the “putative” pore.

**FIGURE 4:**
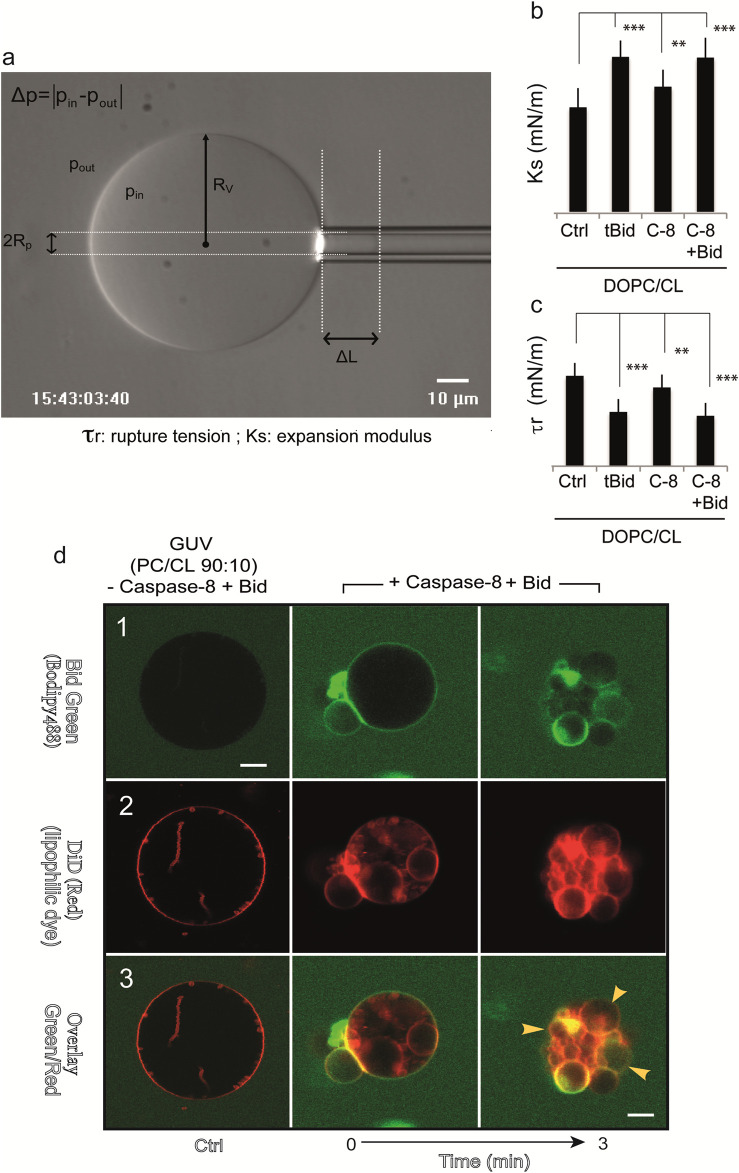
Mechanical properties of GUV vesicles when caspase8 is activated and BID is added. **(a)** Determination of the micromechanical properties of giant unilamellar vesicles (GUVs) via microaspiration. (a) Videomicrograph of a vesicle aspirated in a glass suction capillary. The main variables used to determine the area expansion modulus are as follows: RV, the vesicle radius; pin and pout, the pressure inside and outside the vesicle; and DL, the length of the membrane meniscus inside a glass pipette with an inner radius Rp. Excess membrane tension t is generated by suction such that △p≠0. (b,c) Histograms of the micromechanical quantities measured in the test system under different experimental conditions. **(b)** Ks: Young’s modulus (mN/m); **(c)** τr: Tensile strength (mN/m). Caspase-8 was added to a final concentration of 290 nM, tBID was added to 30 nM, and BID was added to 50 nM. Fisher’s test was used for statistical analysis of differences for both Ks and tr measurements (50 nM. Fisher’s test was used for statistical analysis of differences for both Ks and tr measurements (**, p = 0.01 and***p = 0.05). **(d)** Confocal microscopy study of BID and caspase-8 binding to giant unilamellar vesicles containing cardiolipin. Trios of images (top, middle and bottom) for the same sample: two images obtained with two different detector channels of the microscope, together with an overlay image. DOPC/CL (90:10) vesicles are shown in panels (1 - 3). Top: in (1), protein binding to GUVs is shown in green (this binding becomes apparent only when the green label accumulates at the membrane); middle: the GUV membrane was labeled with 0.05% of the hydrophobic dye DiO, as shown in (2); bottom: overlay of green and red images (3). The time is given in minutes. The arrows indicate a decrease in GUV fluorescence following the formation of a complex between procaspase-8 and BID-Alexa488, resulting in a nonfluorescent tBID. Schematic interpretation and confocal images are reproduced with permission from (Lutter et al., 2000).

Another model system has proven useful, consisting of giant unilamellar vesicles (GUVs) composed mainly of DOPC and CL, with CL contents ranging from 0% to 20% (mol/mol) (Jalmar et al., 2010), to mimic part of the lipid composition of the contact site. The GUVs allow us to assemble an *in vitro* platform consisting of recombinant caspase-8, BID and cardiolipin, which form an activation device on the surface of giant unilamellar vesicles (Figure 4a). This simplified system was used to test the peptides that make up the BID molecules. An original study investigated the mechanism by which tBID interacts with mitochondria released from mouse hepatocytes and disrupts mitochondrial function. The helix α H6 is responsible for the primary targeting of tBID to the mitochondrial CL and the disruption of mitochondrial bioenergetics. Specifically, αH6 interacts with mitochondria through electrostatic interactions involving positively charged lysines 157 and 158. The αH6 helix inhibits state 3 respiration and uncouples state 4 respiration (Gonzalvez et al., 2010). Thus, tBID requires its helix αH6 to efficiently induce cytochrome c release and apoptosis. The optimal sequence for binding is the combination of helices αH6-H7-H8, whereas the BH3 domain is essential for interaction with BAX/BAK and BCL-2. GUVs are quite useful for membrane binding studies (Jalmar et al., 2010) and are susceptible to analysis by flow cytometry. This capability has led to the establishment of a new flow cytometric technique to test the disruption and re-vesiculation of GUVs at smaller vesicle sizes under the action of fully activated caspase-8 and tBID generation (Jalmar et al., 2013). The *in vitro* system used, which mimics contact sites and/or cardiolipin-enriched microdomains on the outer mitochondrial surface, is quite similar to the system described above (Jalmar et al., 2010). Vesicles were analyzed by confocal microscopy or flow cytometry. The requirement of intact mature cardiolipin for caspase-8 activation, FL-BID binding and cleavage, and tBID action was demonstrated (Figure 4b). Both flow cytometry analysis and confocal microscopy confirmed the disruption of the initial vesicles and their re-vesiculation to smaller sizes (Jalmar et al., 2013) due to changes in membrane curvature after the activation of caspase-8, active delocalization of activated caspase-8 and BID to the mitochondrial MOM and BID binding to it (Jalmar et al., 2013) (Figure 4b). These results, together with the initial work of Sorice et al. (2000) and Sorice et al. (2004) and the subsequent work of Jalmar et al. (2010) and Jalmar et al. (2013), confirm that “cardiolipin-enriched microdomains” exist at the MOM (mainly at the contact site or if extended and MICOS), which form activating platforms for the efficient transduction of apoptotic signals on mitochondria (Gonzalvez et al., 2008; Schug et al., 2011). Biophysical approaches, including Laurdan fluorescence and rupture/tension measurements (Figures 4a–c), were used to determine the ability of these three components (cardiolipin, caspase-8 and BID) to meet the minimum requirements for the formation and activity of a functional platform at the surface of the mitochondrial membrane (Gonzalvez et al., 2008).

### 2.4 Did cytochrome *c* play a role in CL and CLOOH movements from the MOM to the IMM contact sites?

Cytochrome *c* (dissociation from the IMM, movement into the intermembrane space, and subsequent release into the cytosol are recognized as key early events in the intrinsic (mitochondrion-initiated) pathway of oxidative stress-induced apoptosis (Korytowski et al., 2011; Raemy and Martinou, 2014; Choi et al., 2007).

Princep’s work has shown that CL deficiency (Choi et al., 2007) or CL peroxidation (Kriska et al., 2005) reduces cytochrome *c* interactions with the inner mitochondrial membrane and facilitates the release of cytochrome *c* into the cytosol after apoptosis stimulation (Asumendi et al., 2002).

Numerous studies over the past decade have demonstrated that CL and BCL-2 homology (BH) domain proteins, such as BH3-only BID and BAX (or BAK), play key roles in the intrinsic (mitochondrion-initiated) pathway of oxidative stress-induced apoptosis (Korytowski et al., 2011; Kriska et al., 2005; Asumendi et al., 2002).

Recent data from fluorescence resonance energy transfer experiments have shown that membrane permeabilization by tBID and BAX requires ordered uptake and interaction of these proteins. BCL-XL antagonizes this interaction by binding tBID (Lovell et al., 2008).

During respiration and oxidative phosphorylation, *cytochrome c* shuttles electrons from complex III to complex IV, but under stress, the interactions are reduced. This affects the relationship between cardolipin and cytochrome *c*, and cytochrome *c* becomes freely available and susceptible to leaving the intermembrane space for the cytosol, where it affects the formation of the caspase-9-activating apoptosome complex (Adrain and Martin, 2001; Zou et al., 1999; Tsujimoto, 1998; Cain et al., 2002; Chinnaiyan, 1999). Cytochrome *c* mobilization has been linked to oxidative modifications of structural IMM lipids, particularly CLs, which usually interact with cytochrome c via hydrophobic and electrostatic interactions (Spooner and Watts, 1992). Owing to its four fatty acyl chains, all unsaturated, natural CLs are much more susceptible to oxidation than phospholipids with two fatty acyl chains are (Spooner and Watts, 1992). Artificial membrane models with CL in thin film or liposomal systems have shown that its normal tight association with cytochrome c progressively weakens with increasing CL peroxidation (CLOOH) (Sen et al., 2006).

Cytochrome *c* (cytochrome c) release upon the oxidation of cardiolipin (CL) from the IMM under oxidative stress occurs early in the intrinsic apoptotic pathway. We postulated that CL oxidation mobilizes not only cytochrome *c* but also CL itself in the form of hydroperoxide species (CLOOH), which translocate from the IMM to the MOM, where it becomes accessible from the cytoplasm (Belikova et al., 2007; Belikova et al., 2006). Indeed, relatively hydrophilic CLOOH could support apoptotic signaling by translocating to the MOM, thus promoting the recruitment of the proapoptotic proteins truncated tBID and BAX for the generation of cytochrome c-traversable pores (Belikova et al., 2006).

CLOOHs can originate from several different sites along the IM and mostly from the outer leaflet sites, where bound cytochrome *c* can function as a CL peroxidase (Belikova et al., 2007; Belikova et al., 2006). The possibility of IMM-to-MOM CLOOH transfer under stress is based on several arguments, i.e., the high concentration of CL at the IMM level, the fact that CL has a high degree of unsaturation, making it available for peroxidation, and finally, the short average distance between the IMM and MOM (50 - 70 angström), together with an initial steep negative CLOOH concentration gradient, which would favor diffusion between the two membranes. In this context, several mechanisms are based on protein-facilitated transfer. One of them is that cytosolic FL-BID has been described as a potential lipid transfer protein (Degli, 2002), such that during mitochondrial phospholipid recycling, it can deliver the CL to extramitochondrial protein targets (caspase-8, tBID or BAX). In addition, oligomeric creatine kinase and nucleoside diphosphate kinase, which reside at contact sites, are thought to facilitate IMM-to-MOM phospholipid transfer with high affinity for CL in a liposomal model (Degli, 2002; Epand et al., 2007). A recent study (Liu et al., 2008) tested the hypothesis that mitochondrial phospholipid scramblase-3 (PLS3) plays a critical role in TNF-α-induced apoptosis of HeLa cells by mediating CL translocation to the mitochondrial surface. This was confirmed by the finding that PLS3 overexpression increased the amount of CL in the MOM, whereas PLS3 knockdown decreased it, with a corresponding change in tBID binding.

### 2.5 tBID-ROS-dependent mitochondrial permeabilization waves

BID and its truncated form tBID intervene in the generation of radicals through tBID interaction with mitochondrial cardiolipin (Lutter et al., 2000). Reactive oxygen species contribute to cell death and caspase activation by promoting FLICE-inhibitory protein degradation and the mitochondrial release of cytochrome *c.* In the latter, superoxide anions do not affect BAK oligomerization but promote mitochondrial crista reorganization and membrane lipid peroxidation. Antioxidants can reverse these changes and thus protect against TNF-α− or anti-Fas-induced apoptosis.

At the interface of apoptosis initiation and execution, MOMP can spread through cells in a spatiotemporal manner, particularly in response to extrinsic apoptosis induction (Garcia-Perez et al., 2012). Some curious mechanisms support this behavior of MOMP, which appears to spread through cells in a wave-like manner (Jacob et al., 2016). Two different mechanisms underpin these observations: a reaction‒diffusion mechanism based on anisotropic anisotropies in the production of the MOMP inducer truncated BID (tBID) and/or a process driving the spread of MOMP mediated by a sequential burst of reactive oxygen species (ROS) (Garcia-Perez et al., 2012). Indeed, the integration of both models into a combined mathematical description of spatiotemporal tBID and ROS signaling accurately reproduced all available data and provided robustness to spatial MOMP propagation when mitochondria are spatially separated.

### 2.6 Mitochondrial membrane curvature

At the molecular level, cell membranes can be thought of as flat structures. With the acquisition of a certain degree of curvature, each monolayer becomes more stable. There are several basic rules, i.e., the minimum curvature energy is associated with a monolayer bending to a degree equal to its intrinsic curvature.

Any bilayer with a large negative intrinsic monolayer curvature spontaneously evolves into an inverted phase, called the hexagonal phase. The phospholipids are then arranged as cylinders with an aqueous core, and the phospholipids are oriented with their head groups adjacent to the aqueous core. Curvature is thought to be important in determining the function of the mitochondrial membrane. Mitochondrial lipids are known to undergo a transition from a lamellar phase to a hexagonal phase in the presence of Ca^2+^ (Zeng and Lin, 1992; Tang et al., 2012). Freeze-fracture electron microscopy of structures in intact mitochondria has demonstrated the presence of nonbilayer structures (Van Venetie and Verkleij, 1982). The propensity to exhibit hexagonal phases has been linked to calcium movements across the membrane (Ortiz et al., 1999) as well as to the control of mitochondrial enzymes (Garcia et al., 2023). Interestingly, cytochrome *c* itself is a known inducer of hexagonal phase formation in the bilayers of cardiolipin (Bergstrom et al., 2013; Pataraia et al., 2014). When using rupture tension/models in GUVs (Figures 4a–c) or flow cytometry analysis, it is evident that the membrane curvature changes linked to the CL are at work, inducing rapid vesicle disruption and possible re-vesiculation at a lower size (Jalmar et al., 2010) (Figure 4d).

### 2.7 tBID and the initial destabilization of mitochondrial bioenergetics

The sequence of events by which BID (FL-BID) and its cleavage product tBID are inserted into the MOM at the contact site (and/or the so-called “MICOS”) is now widely accepted (Kantari and Walczak, 2011; Gonzalvez et al., 2008; Gonzalvez and Gottlieb, 2007; Sorice et al., 2009) in the context of the Fas ligation or TRAlL pathway, which is more or less the prototype of the apoptotic pathway (Figure 1).

More specifically, pro-caspase-8 binds to death effector domains, where it is activated, and then binds to accessible cardiolipin at the MOM, where full activation occurs. The binding and insertion of BID appear to be very precise and involve an ordered sequence of events that primes the mitochondria for the action of BAX and BAK. BID then delocalizes from the cytoplasm to bind fully activated caspase-8 (bound to cardiolipin) and is then cleaved to yield its active counterpart, tBID, which also has a very high affinity for cardiolipin;

1. tBID interacts with mitochondria by specifically binding to cardiolipin (CL) (and later later on with peroxidized cardiolipin (CLOOH) and CL degradation products such as monolysocardiolipin (MLCL)) and immediately disrupts mitochondrial structure and function independently of its BH3 domain
2. tBID then activates BAX and/or BAK through its BH3 domain and induces their subsequent oligomerization in the mitochondrial membrane (Gonzalvez et al., 2010).

Other constructs, such as the hairpin-forming domain αH6-H7 domain, are described. Hydrophobicity was calculated via PROTSCALE software from the Swiss Institute of Bioinformatics according to the methods of Kyte and Doolittle (Kyte and Doolittle, 1982). αH6 and αH6m are shown in italics. The table is taken and slightly modified from (Gonzalvez et al., 2010).

All the constructs are electropermeabilized to allow their penetration into cells. CV-1 cells were transfected with plasmids encoding BID-EYFP, tBID-EYFP or tBID-EYFP mutants in the presence of 10 μM Bok-D. After 24 h, the cells were stained with 20 nM of the mitochondrial potential probe TMRE, and the localization of the tBID-EYFP mutants was determined by calculating the TMRE/EYFP ratio via microspectroflurometry. A ratio of 1.0 indicates that EYFP colocalizes exclusively with TMRE at the mitochondria, whereas a lower ratio indicates that EYFP also localizes to the cytosol. The * marks the constructs that localize to the mitochondria but are unable to induce cytochrome *c* release or alter mitochondrial bioenergetics. The constructs that localize to the cytoplasm are in italics. The table is adapted and slightly modified from (Gonzalvez et al., 2010).

**FIGURE 5:**
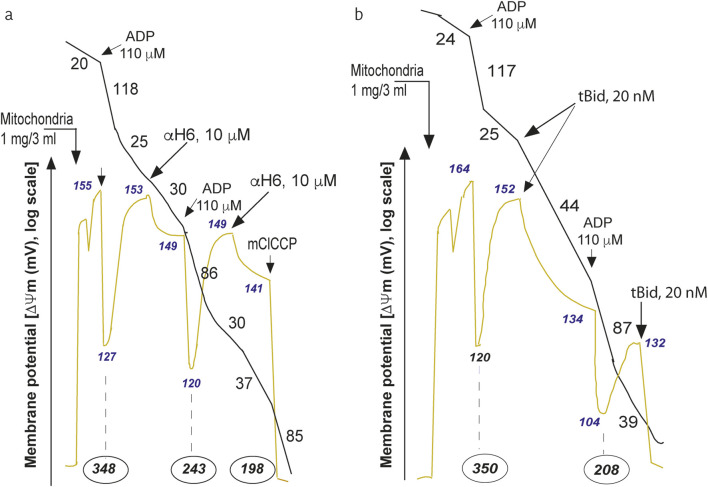
Mitochondrial bioenergetic changes induced by the a-H6 helicoidal peptide derived from tBID or by a recombinant tBID molecule. The α H6 helix affects mitochondrial bioenergetics. Purified mouse liver mitochondria (M) were incubated in respiratory buffer (33 μg/mL). Oxygen consumption (V_oxidation_, black line) and the mitochondrial potential (ΔΨm, blue line) were monitored via a Clark-type electrode coupled to a tetraphenylphosphonium (TPP^+^) cation-sensitive electrode as described previously (Gonzalvez et al., 2010). Succinate (10 mM) was added as an oxidizable substrate (1 mM sulfonate). ADP was added at 110 μM, and mClCCP was added at 10 μM. The numbers along the traces represent the values of the oxidation rates in nmol O_2_/min/mg protein (black), the mitochondrial potential in mV (yellow/green) and the number given in black (encircled by an oval), which is expressed in nmol ATP produced/min/mg protein (Gonzalvez et al., 2010). **(a)** Effects of the addition of the aH6 peptide derived from the tBID protein (10 mM) to the purified mitochondria on the oxygen consumption ΔΨm and proton efflux (nmol ATP produced/min/mg protein). **(b)** Effects of the addition of recombinant tBID (20 nM) to purified mitochondria on oxygen consumption ΔΨm and proton efflux (expressed in nmol ATP produced/min/mg protein). The experiment was performed by Gonzalvez F., Diolez P. and P.X. Petit and was based on a series of experiments that have served to illustrate the following published manuscript (Gonzalvez et al., 2005a).

The underlying mechanisms responsible for destabilization of mitochondrial bioenergetics are described below. As previously described, tBID is widely inserted into the mitochondrial membrane. Extensive work with artificial peptides (Tables 1, 2) issued from the tBID structure revealed that the tBID αH6 helix is the minimal domain able to resume binding to the MOM. Its binding evolved through electrostatic interactions involving the positively charged lysines 157 and 158 of the αH6 helix (Figure 5a) and mostly recapitulates the effect of tBID on mitochondrial bioenergetics (Figure 5b). The binding of αH6 leads to the inhibition of ADP-stimulated respiration (state 3 respiration), which is associated with a slight uncoupling of state-4 respiration (Gonzalvez et al., 2010). This finding is consistent with the findings of Liu et al. (Liu et al., 2004), who reported that the ability of the BID’s ‘‘putative cardiolipin-binding domain’’, containing αH6, to affect mitochondrial respiration. Biophysical analyses revealed that, like tBID (Gonzalvez et al., 2005a), αH6 binds specifically to CL-enriched lipid monolayers via electrostatic interactions and reorganizes them into microdomains (Gonzalvez et al., 2005a; Gonzalvez et al., 2010).

**TABLE 1:** Somes physical characteristics of Bid, tBid and tBid mutants. Various properties of the different peptide-derived from tBid, i.e., number of amino-acids, isoelectric point (pI), hydrophobility and electric charge.

Names	AA number	pI	Hydrophobicity	Charge +/−
Bid Full-length	193	5.27	−0.488	−6.5
Bid N-ter	60	8.36	−0.579	+ 3
H3	21	5.57	−0.848	−0.5
H4	10	10.55	+ 0.930	+ 1
H5	18	3.90	−1,094	−3
H6	**18**	**10.50**	**+ 1.265**	**+ 2.5**
H7	14	7.55	−0.057	+ 0.5
H8	10	12.2	−1.000	+ 3
H6m	**18**	**7.56**	**+ 1.456**	**+ 0.5**
H6 + ½ H7	27	9.99	+ 0.807	+ 3
H6 + H7	35	9.99	+ 0.650	+3
H6 + H7 + H8	47	10.93	+ 0.212	+5
H4 + H5 + H6	58	9.19	−0.340	+ 1.5
BH3 Domain	25	7.55	−0.596	−0.5

The bold values relate to the most important helices (H6 and H6m) that are indeed localized to the mitochondrial membrane.

**TABLE 2:** Somes physical characteristics of Bid, tBid and tBid mutants. Intracellular localization of Bid-EYFP, tBid-EYFP, and its deletion mutants.

Construct	EYFP/TRME	Localization
tBid	**1** ± 0.07	**Mitochondrial**
tBidΔ94	**0.58** ± 0.04	Cytoplasmic
tBidΔH6	**0.52** ± 0.05	**Cytoplasmic**
tBidΔH6,ΔH7	**0.30** ± 0.04	Cytoplasmic
tBidΔH6, ΔH7, ΔH8	**0.27** ± 0.03	Cytoplasmic
tBidΔΒΗ3*	**1** ± 0.07	Mitochondrial
tBidΔΒΗ3 ΔΗ4−5*	**1** ± 0.07	**Mitochondrial**
tBidΔΒΗ3 ΔΗ6−8	**0.27** ± 0.03	Cytoplasmic

The bold values relate to the most important constructs that are indeed localized to the mitochondrial membrane. ^*^Is pointing the constructs that are still mitochondrially localized.

**FIGURE 6:**
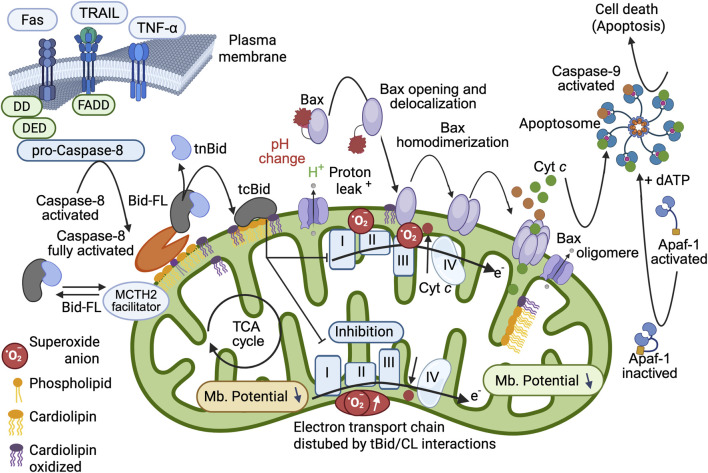
Schematic interpretation of the mitochondrial changes induced by full Caspase-8 activation, cardiolipin binding of BID and tBID insertion into the MOM. Refined model of the pro-apoptotic function of tBID that sets the importance of tBID/CL interactions. First, BID binds to the CL present at the contact site (or MICOS) via its charged α-helices (see Figure 2) and destabilizes the mitochondrial membrane. The interaction of tBID with CL (i.e., following the interaction of preactivated caspase-8 with CL) disrupts the organization of the mitochondrial membrane at the contact sites (and certainly throughout the entire IMM). The induced structural changes, as well as the immediate generation of peroxidized CL, disrupt the activity of the electron transport chain complexes (reduced electron transport rate) and lead to cytosolic acidification, mitochondrial ROS production, and total mitochondrial lipid peroxidation. This environment can prime the activation of BAX and/or BAK and allow their activation and delocalization (with first homodimerization and then oligomerization at the membrane to form a pore). Moreover, tBID interacts through its BH3 domain with BAX and/or BAK and enhances membrane anchorage, which results in the liberation of cytochrome *c* to form apoptosomes in the cytoplasm with dATP, APAF-1 and caspase-9, which become activated and act as the main executioner caspases in cell death. This schematic interpretation was drawn partly with the Scientific Image and Illustration Software/Biorender and is inspired by (Gonzalvez et al., 2005a) but remains under the copyrigth of @Patrice X. Petit.

With respect to the behavior of tBID and its important role in destabilizing mitochondrial bioenergetics, the use of recombinant tBID proteins and microinjection technology allows us to demonstrate that tBID binding to the MOM inside the cell also provokes an inhibition of ADP-stimulated respiration as well as an uncoupling of state 4 respiration (Figure 5b) (Gonzalvez et al., 2005a). These events are accompanied by a significant decrease in ΔΨm [measured in mV, with a tetraphenyl-phosphonium (TPP^+^)-based electrode)] and a reduction in phosphorylation (nmole ATP/min/mg of mitochondrial protein, derived from the measure of proton efflux with a pH electrode) (Figure 5b) (Gonzalvez et al., 2005a). The whole sequence of events at work at the mitochondrial level are depicted in a schematic interpretation (Figure 6).

### 2.8 VDAC2, tBID and BAK: still an intricate situation

The mitochondrial outer membrane voltage-dependent anion channel (Mannella and Kinnally, 2008), also known as the “mitochondrial porin”, has long been implicated in regulating the mitochondrial response to certain cell death stimuli (Galluzzi and Kroemer, 2007). This includes a possible role in MOM permeabilization during apoptosis, which is a key event leading to cytochrome *c* release as a critical cofactor for further activation of caspases. Although controversial, it is possible that VDAC is a component of the permeability transition pore complex (Zheng et al., 2004); therefore, its influence may extend to the inner membrane, regulating the membrane potential and ATP production. Among the strongest indications that VDACs play a role in the regulation of cell death are the reported interactions between VDACs and members of the BCL-2 protein family. The multi-BH domain-containing proapoptotic members BAX and BAK are the essential gateways to MOMP, and it has been suggested that both physically interact with VDACs (Galluzzi and Kroemer, 2007; Kroemer et al., 2007). A minor VDAC isoform, known as VDAC2, physically restrains BAK at the MOM in unstimulated viable cells (Cheng et al., 2003). Notably, in addition to BAX, another proapoptotic protein, BID, which is cleaved with caspase-8 (leading to tBID generation), affects the voltage gating of VDACs by inducing channel closure (Roy et al., 2009).

However, after death, tBID inactivation of BAK by VDAC2 promoted BAK oligomerization. We speculate that by decreasing the probability of VDAC opening, tBID reduces metabolite exchange between mitochondria and the cytosol, leading to mitochondrial dysfunction. PG significantly enhances VDAC oligomerization in the membrane, whereas CL disrupts VDAC supramolecular assemblies. A situation in which VDAC is preferentially inserted into CL-rich domains (Rostovtseva et al., 2006). During apoptosis, the level of PG in mitochondria increases, whereas the CL level decreases. We suggest that the specific lipid composition of the outer mitochondrial membrane might be crucial and, thus, a potential cue for regulating the oligomeric state of VDACs (Betaneli et al., 2012).

Given this evidence, interesting results from gene deletion studies have shown that all three VDAC gene products are dispensable for mitochondria-dependent cell death in mouse embryonic fibroblasts (Baines et al., 2007). In addition, necrotic cell death is caused by defects in the permeability transition pore, e.g., induced by oxidative stress or calcium. A major consequence of VDAC2-mediated recruitment of nascent BAK to mitochondria is the spatial separation of mitochondrial BAK and cytosolic BAX, opening the possibility for differential regulation of these otherwise redundant effectors of MOMP. In addition to VDAC2, BAK has been reported to constitutively interact with MCL-1 in the MOM of viable cells (Cuconati et al., 2003; Nijhawan et al., 2003). These and other interactions are likely to be in equilibrium and may shift toward interaction with tBID depending on relative protein abundance and interaction affinities. One could still speculate that CL and tBID play distinct roles in this context, which has not been fully elucidated.

It is likely that mitochondrial BAK follows the same sequence of activation by tBID and antagonism by pro-surviving BCL-2 members as described for BAX. Like BAX, tBID induces the conformational activation of monomeric BAK ((Ruffolo and Shore, 2003). BCL-2 and possibly BCL-XL, which can sequester tBID, also selectively interact with tBID-activated BAK, preventing its auto-oligomerization and MOMP (Ruffolo and Shore, 2003). However, owing to the spatial separation of BAK and BAX in viable cells, there are likely other mechanisms that prevent BAK or BAK from triggering MOMP and cell death.

## 3 Some news players enter the game together with tBID

### 3.1 The ATR protein kinase is a new player in the game

The phosphatidylinositol 3-kinase (PI3K)-like protein kinase ATR (*Ataxia telangiectasia* and Rad3-related) is central for the maintenance of genome integrity in the context of DNA damage (Cimprich and Cortez, 2008; Zeman and Cimprich, 2014; Sancar et al., 2004) and is also a key protein that prevents the onset of cancer (Bartkova et al., 2005). ATR is a checkpoint kinase that phosphorylates hundreds of downstream proteins during DNA damage responses (Matsuoka et al., 2007). ATR acts as a complex with ATR-interacting protein (ATRIP), which senses replicative stress-induced DNA damage, activates checkpoints, arrests the cell cycle and facilitates repair to restore DNA integrity (Cortez et al., 2001).

**FIGURE 7:**
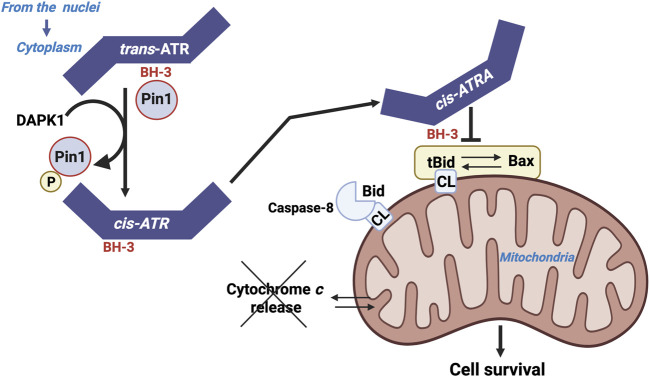
ATR as a BH3 only domain that interact with tBID. Localization of ATR to mitochondria may occur either through the binding of ATR-H to mitochondria-bound tBID or through cytoplasmic ATR-H-tBID interactions to facilitate mitochondrial localization of the ATR-H-tBID complex.

ATR has a BH3-like domain that allows it to interact with the tBID counterpart of Bid. ATR then exerts an antiapoptotic effect similar to that of BCL-2 and BCL-XL (Hilton et al., 2015). This activity is patent in the context of UV-induced apoptosis (Figure 7).

The action of ATR appears to be independent of its hallmark checkpoint and kinase activities, and it does not involve its usual partner, ATRIP. The antiapoptotic action of ATR is mediated by its interaction with tBID at the mitochondrial membrane, which blocks cytochrome *c* release from the intermembrane space and subsequent apoptosis.

Pin1 (a peptidylprolyl *cis*/*trans* isomerase NIMA-interacting) downregulates the activity of ATR localized to the mitochondria by isomerizing ATR from the cis-isomer to the trans-isomer at the phosphorylated Ser428-Pro429 motif. UV exposure inactivates Pin1 via the Death Associated Protein Kinase 1 (DAPK1, which belongs to a family of five serine/threonine (Ser/Thr) kinases that possess tumor suppressive functions and mediate a wide range of cellular processes, including apoptosis and autophagy), which stabilizes the pro-survival cis-isomeric ATR (Hilton et al., 2015; Singh et al., 2016). The unexpected cytoplasmic response of ATR undoubtedly underlies the observed antiapoptotic role of ATR in suppressing carcinogenesis, as well as its inhibitory action in sensitizing anticancer agents to kill cancer cells. This information highlights the fact that tBID emerges as a pivotal determinant that dictates the cell fate between apoptosis and carcinogenesis (Makinwa et al., 2024).

### 3.2 ATM kinase is also a player in the game together with tBID

Several studies have reported that BID is phosphorylated by the protein kinase ataxia telangiectasia mutated (ATM) and that BID plays a protective role during DNA damage (Zinkel et al., 2005; Kamer et al., 2005; Maryanovich et al., 2012; Tasdogan et al., 2016). There is some controversy regarding *in vitro* studies (Cao et al., 2007); however, the fact that non-phosphorylated BID-S61A/S78A-knock-in mice are more sensitive to IR-induced death (Maryanovich et al., 2012) is evidence that BID, via its activities in the mitochondria, is a pivotal regulator of the DNA damage response *in vivo*.

### 3.3 MTCH2 as a facilitator

MTCH2 indeed acts as a facilitator of tBID recruitment to mitochondria, but that is not the only/main function of MTCH2. Many publications have demonstrated that MTCH2 essentially plays a critical role in regulating lipid metabolism and mitochondrial dynamics (Bar-Lev et al., 2016; Buzaglo-Azriel et al., 2016; Rottiers et al., 2017; Bahat et al., 2018; Goldman et al., 2024; Chourasia et al., 2025; Zhao et al., 2025). Additionally, several studies have reported that BID plays a role in regulating obesity/metabolism (Ni et al., 2010; Yan et al., 2022). On the basis of these and other studies mentioned in this review regarding cardiolipin and respiration, it is tempting to speculate that BID regulates metabolism, obesity, and respiration by interacting with and regulating MTCH2 activity. Furthermore, these studies suggest that BID/MTCH2 may regulate metabolism and may be involved in MOMP.

MTCH2 has been reported to function as both an MOM insertase (Guna et al., 2022) and a phospholipid scramblase (Guna et al., 2022). VDAC also functions as a phospholipid scramblase (Zhao et al., 2025; Bartos et al., 2024). Upon reconstitution into membrane vesicles, dimers of human VDAC1 and VDAC2 rapidly translocate phospholipids across the bilayer via a mechanism unrelated to their channel activity. Thus, it is tempting to assume that, *in vivo*, VDAC isoforms—members of a superfamily of beta-barrel proteins—moonlight a class of phospholipid scramblases distinct from the alpha-helical scramblase proteins that import lipids into mitochondria. Since BID has been described as a potential lipid transfer protein (Degli, 2002), it is possible that this activity involves cooperation with VDAC and/or MTCH2. Once again, the regulation of lipid composition and protein insertion into the MOM may play a role in MOMP.

### 3.4 Humanin as an inhibitor that sequesters tBID into fibre structures

A wide class of mitochondrial retrograde signaling peptides, known as mitochondrial-derived peptides (MDPs), has been described for its ability to interact with BCL-2 proteins endogenously. They all affect MOMP-mediated apoptosis (Guo et al., 2003; Zapala et al., 2010). Humanin (HN) is a natural short peptide that protects cells against various stress conditions and apoptosis. More precisely, Humanin is a mitochondrial-derived peptide that is expressed from an alternate ORF in the mitochondrial genome and that is susceptible inhibiting apoptosis through interactions with the pro-apoptotic BCL-2 proteins (i.e., tBID or BAX). It has been recently reported that BAX sequestration and inactivation by HN result in their mutual reformation into β-sheet fibers *in vitro* (Morris et al., 2019). Concerning BID, HN also appears to initiate the generation of fibers. The use of a biophysical approaches (with spectroscopic techniques, mass analysis) associated to protein fragmentation and electron microscopy allows to validate the formation of fiber structure when HN and Bid interacts. Moreover, β-sheet structures can be observed as well as fibrillate (Morris et al., 2020). The fibers exhibit uniform diameter with BID situated in the fiber core. Ligth scattering experiment under various conditions illustrate the sensitivity for fiber formation to environmental conditions (i.e., mitochondrial outer membrane vinicity). This suggests that the interaction between BID and HN, which promotes fibre formation, may be modulated by intracellular pH, temperature and membrane localization. Since HN is also susceptible to interacting with MCL-1, peptides in this family are being investigated intensively in the hope that targeting them will lead to fruitful results for therapeutic uses.

## 4 Multiple entanglements of different cell death pathways in which Caspase-8 and BID caught in the act with cardiolipin

### 4.1 BID is the missing link between pyroptosis and classical mitochondrially induced cell death (apoptosis)

**FIGURE 8:**
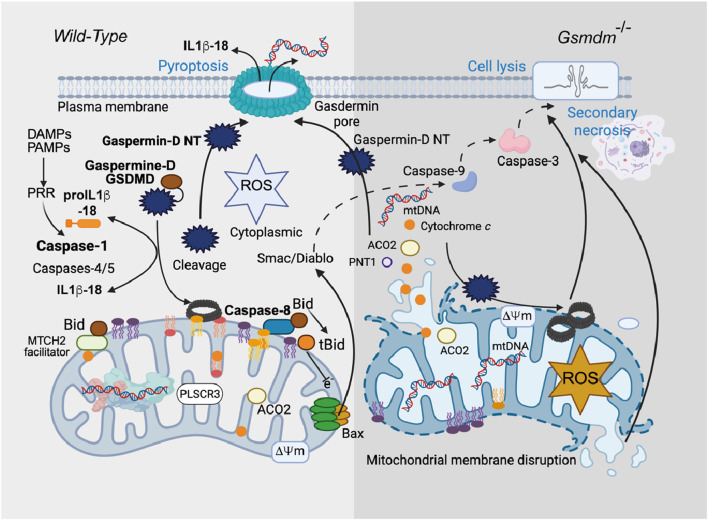
Gasdermine, mitochondria, necroptosis and pyroptosis. Gasdermin D (GSDMD)-induced inflammatory cell death (pyroptosis) causes mitochondrial damage. Although gasdermins are known to be involved in pyroptosis, the complex mechanism that leads to cell death remains complex and needs to be mapped. In a recent work, Miao et al. (2023) reported that the mitochondrial compartment is critically involved and represents a “point of no return” in inflammatory processes and subsequent cell death. Cardiolipin binds gasdermins that form pores at the mitochondrial membrane level that disrupt both mitochondrial bioenergetic homeostasis and the inhibition of oxidative phosphorylation, superoxide formation at complex II, and the release of cytotoxic mediators (e.g., cytochrome *c* and Smac/DIABLO). The hypothesis of BID/tBID activation is also probable (Gonzalvez et al., 2008). In parallel, mitophagic processes are at work, in addition to the ability of gasdermin to form other pores at the cytoplasmic membrane surface and promote a necrotic type of death.

Pyroptosis (Kepp et al., 2010) is a type of lytic inflammatory cell death driven by caspase-1 cleavage of Gaspermine-D (GSDM-D) (Cerretti et al., 1992; Shi et al., 2017). Caspase-1 is involved in the activation of proinflammatory cytokine genes (pro-IL-1β and pro-IL-18) that express the IL-1β and IL-18 proteins, which are commonly called IL-1 converting enzymes because of their activity (Heilig et al., 2020; Black et al., 1989) (Figure 8). Caspase-1 also induces inflammation-induced cell death or a lytic form of programmed cell death called pyroptosis through proteolytic activation of Gasdermine D (Shi et al., 2017). The cell lysis that occurs in GSDM-D-deficient cells upon activation of canonical inflammasomes is a rapid form of secondary necrosis and is dependent on caspase-1-dependent activation of either caspase-8 or caspase-9, BID cleavage (T et al., 2019), SMAC and cytochrome *c* release, and caspase-3 activity (Wang and Tjandra, 2013). Secondary necrosis describes the loss of membrane integrity of apoptotic cells or apoptotic bodies and is thus appropriate because death in GSDM-D-deficient cells is dependent on initiator caspases and executor caspase-3 and results in loss of membrane integrity. However, it is remarkably different from normal apoptosis in terms of the signaling pathways underlying its induction, the cellular morphology, and the accelerated rate at which cells die.

Curiously and surprisingly, in the absence of GSDM-D (T et al., 2019), only delayed cell lysis occurs, which promotes the expression of caspase-1, a key regulator of alternative cell death pathways. In the absence of GSDM-D, caspase-1 functions (Figure 8), activating initiator and executioner caspases and triggering rapid progression to necrosis. This pathway requires direct caspase-1-driven truncation of BID (Neitemeier et al., 2017), tBID insertion into the mitochondrial membrane, and possibly direct generation of caspase-3 p19/p12 by either caspase-8 or caspase-9 (T et al., 2019). It seems quite clear that BID is directly cleaved by caspase-1 in this system (Figure 8). Induction of tBID allows permeabilization of the MOM, which is required in this system to release SMAC/diablo and relieve inhibitor of apoptosis protein inhibition of caspase-3, thereby allowing caspase-3 autoprocessing to the fully active p17/p12 form (Neitemeier et al., 2017).

These signaling sequences are quite remarkable and suggest that cell lysis in inflammasome-activated GSDM-D-deficient cells is related to strong synergy between rapid caspase-1-driven activation of both initiator caspase-8/-9 and BID cleavage (Heilig et al., 2020; Neitemeier et al., 2017). This phenomenon, which appears to be an amplification of the mechanism, leads to rapid activation of caspase-3 and an immediate transition to so-called secondary necrosis (Yin et al., 1999). In terms of host‒pathogen interactions, this system may be advantageous for the host to counteract pathogen-induced inhibition of GSDM-D and may play a role in the use of GSDM-D inhibitors in immunotherapies for caspase-1-dependent inflammatory disease.

On the other hand, it has recently been established that the N-terminus (GSDM-D) also initiates the formation of mitochondrial pores through a direct relationship with oxidized cardiolipin. This event could be related to the generation of mitochondrial reactive oxygen species (ROS) during endotoxemia. Subsequently, caspase-4/11 initiates GSDMD permeaSbilization, which is amplified by the upregulation and activation of NLRP3 inflammation through further generation of ROS. GSDMD-N pores form prior to BAX-induced MOMP and further initiate BAX oligomerization and exacerbate secondary necrotic processes (Tang et al., 2024). Indeed, oxidized cardiolipin is the ultimate target of GSDMD in cardiomyocyte mitochondria during endotoxin-induced myocardial dysfunction (EIMD), and the modulation of cardiolipin oxidation may be a therapeutic target early in the disease process to prevent EIMD (Tang et al., 2024).

### 4.2 BID cleavage drives the crosstalk between pyroptosis agent-induced ER stress and TRAIL-induced apoptosis

Among the different subtypes of programmed cell death, including canonical apoptosis, ferroptosis, and cuproptosis, pyroptosis (Ketelut-Carneiro and Fitzgerald, 2022) was originally described as the caspase-1-dependent cell death of *S. typhimurium*-infected macrophages.

Here, the pro-inflammatory nature of pyroptosis was recognized immediately, and the immunogenic cell death modality was accepted (Ghiringhelli et al., 2009). Activation of caspase-1 (‘‘IL-1β-converting enzyme’’) leads to the release of the pro-inflammatory cytokines IL-1b and IL-18 and occurs before any other morphological characteristic of cell death (Brough and Rothwell, 2007). Notably, pores are rapidly formed in the plasma membrane of pyroptotic cells, with cytoplasmic swelling resulting in osmotic lysis through plasma membrane disruption (Fink and Cookson, 2006). The formation of these pores reportedly requires caspase-1 activity as well as the rearrangement of the host cell actin cytoskeleton (Fink and Cookson, 2006). These pores provide inflammatory molecules, including (but presumably not limited to) IL-1β and IL-18 (both of which are generated within the cytosol) (Brough and Rothwell, 2007), with a direct route for release, and may therefore be instrumental for the pro-inflammatory character of pyroptosis. While pyroptotic DNA fragmentation has been shown to occur independently of caspase-activated DNase (Fink and Cookson, 2006), it remains to be determined whether caspase-independent nucleases such as apoptosis-inducing factor or endonuclease G might be involved in chromatolysis (Kroemer et al., 2007). Therefore, pyroptosis shares morphological features of both apoptosis and necroptosis (Ketelut-Carneiro and Fitzgerald, 2022).

With respect to BID truncation events, when HCT116 (human colorectal carcinoma) cells are treated with TRAIL in conjunction with erastin (Hou et al., 2023) and artesumate (Jiang et al., 2021), which induce ferroptosis, TRAIL classically enhances caspase-8 activation, and BID truncation increases in parallel. When ovarian adenocarcinoma OVCAR-3 cells were treated in the same way, similar caspase-8 activation and tBID production were observed. When BID mutants are used, the mitochondrially induced apoptotic pathway is not activated. These findings indicate that BID plays a key role in the cross-talk between TRAIL-induced apoptosis and the ER stress response, leading to ferroptosis (Kim et al., 2022).

### 4.3 The curious behavior of FL-BID and t-BID during verotoxin-1-induced apoptosis in Burkitt’s lymphoma cells

Among tumor cells, globotriaosylceramide (Gb3) is highly expressed in Burkitt’s lymphoma (BL) cells as well as in other solid tumors, including breast, testicular and ovarian carcinomas. Gb3 is a glycosphingolipid expressed on a subpopulation of germinal center B lymphocytes and has been recognized as the B-cell differentiation antigen CD77 on a subpopulation of germinal center B lymphocytes.

Verotoxin-1 (VT-1), a Shiga toxin expressed by enterohemorrhagic *Escherichia coli* and enteric *Shigella dysenteriae*-1 pathogens, is a known ligand of the cell surface Gb3/CD77 complex (Unsay et al., 2017). To poison cells, the toxin A moiety must be cleaved by furin and transported retrogradely to the Golgi apparatus and endoplasmic reticulum, where the enzymatically active part of the A moiety is translocated to the cytosol, where it inhibits protein synthesis and can induce apoptosis.

VT-1 induces nearly “prototypical apoptosis” via a caspase-dependent and mitochondria-dependent pathway. The apoptotic pathway was found to act through the cleavage of the BID protein by caspase-8 to generate t-BID-activated BAK and BAK. However, it appeared that t-BID did not play its usual role with respect to BAK and BAX. Their activation occurred under the control of BID. Experiments in which a non-cleavable form of BID (BID-D59A) was introduced into BID-deficient BL cells revealed that BAK and BAX activation were restored after VT-1 treatment (Debernardi et al., 2018). However, tBID is still involved in the cytosolic release of cytochrome *c* from the mitochondrial intermembrane space, as is BAX/BAK. BID was found to control the homo-oligomerization of both BAK and BAX, likely contributing to the initial small release of cytochrome *c,* whereas t-BID was required for further hetero-oligomerization, which controls the amplification of cytochrome *c* release. In this model, the functional cooperation between BAK and BAX during VT-1-induced apoptosis and, unexpectedly, the activation of caspase-8 and the production of t-BID were not initially mandatory for the cell death process, although all these events are required for the full completion of the cell death pathway.

### 4.4 tBID, Beclin-1 and autophagy

**FIGURE 9:**
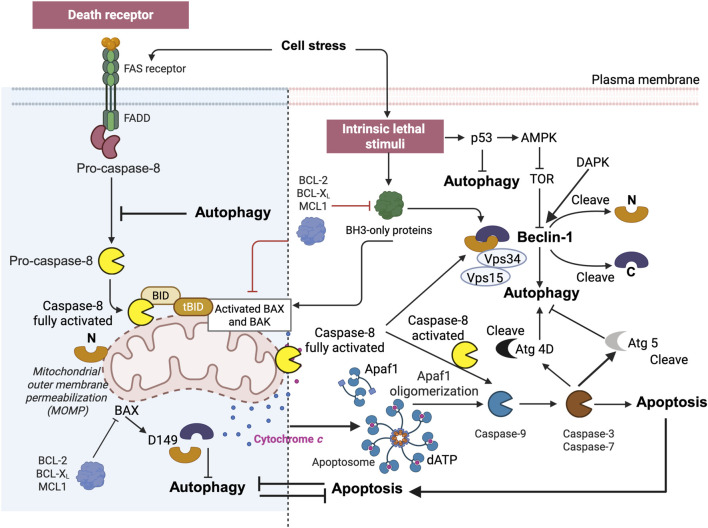
BID and tBID are also at the intersection of apoptosis and autophagy. The complex interplay between apoptosis and autophagy. Apoptosis can be triggered by external, receptor-dependent stimuli or by internal, mitochondria-mediated signaling. Moreover, apoptosis shares common stimuli and signaling pathways and exhibits some degree of mutual inhibition. During sustained exposure to apoptotic stimuli, caspase-mediated cleavage of Beclin 1 results in the generation of fragments (“N” and “C”) that lose their ability to induce autophagy. The C-terminal fragment translocates to the mitochondria and sensitizes cells to apoptotic signals. Although the apoptosis-associated cleavage of Beclin 1 and Atg5 inactivates autophagy, the cleavage of Atg4D by caspase-3 generates a fragment with increased autophagy activity. Moreover, autophagy inhibits apoptosis partly by degrading active caspase-8 or preventing the activation of BID by Beclin 1. The figure is directly inspired by the work of Kang et al. (2011) and Kang and Tang (2017).

Crosstalk between apoptosis and autophagy. Autophagy and apoptosis share common stimuli and pathways and exhibit a degree of mutual inhibition. During prolonged exposure to apoptotic stimuli, caspase-mediated cleavage of Beclin-1 results in the generation of fragments (‘N' and ‘C') that lose their ability to induce autophagy (Kang et al., 2011) (Figure 9). The C-terminal fragment translocate to the mitochondria and sensitizes cells to apoptotic signals. Although the apoptosis-associated cleavage of Beclin-1 and Atg5 inactivates autophagy, the cleavage of Atg4D by caspase-3 generates a fragment with increased autophagy activity. In addition, autophagy inhibits apoptosis in part by degrading active caspase-8 or by preventing the activation of BID by Beclin-1 (Grohm et al., 2010).

### 4.5 BID at the crossroad of “ferroptosis” oxytosis)

Oxidative cell death may be mediated by a variety of secondary routes, such as oxytosis (Tan et al., 2001) and ferroptosis (Xie et al., 2016). Specifically, oxytosis was initially characterized in 1989 as a calcium-dependent form of nerve cell death induced by glutamate.

Oxytosis, formerly known as glutamate oxidative toxicity, is a form of regulated nonapoptotic cell death associated with the accumulation of reactive oxygen species, the depletion of intracellular glutathione, and the impaired absorption of cystine by the amino acid antiporter XC-. Numerous studies have focused on this form of glutamate toxicity using a specific hippocampal neuronal cell line (HT-22 cells) selected for its sensitivity to glutamate (Tan et al., 2001). Oxytosis is associated with age-related disorders (e.g., Alzheimer’s disease) and other pathological conditions.

In contrast, ferroptosis is characterized as an iron-dependent form of oxidative cell death. It is induced by the accumulation of lipid peroxides. Ferroptosis shares several similarities with oxytosis, including the inhibition of cystine uptake by system Xc-, glutathione depletion, and subsequent inactivation of glutathione peroxidase 4 (GPX4). Unlike the term “oxytosis,” which was used primarily in early studies of neurodegenerative diseases, the term “ferroptosis” has been widely used in cancer and noncancer diseases, including neurodegeneration.

**FIGURE 10:**
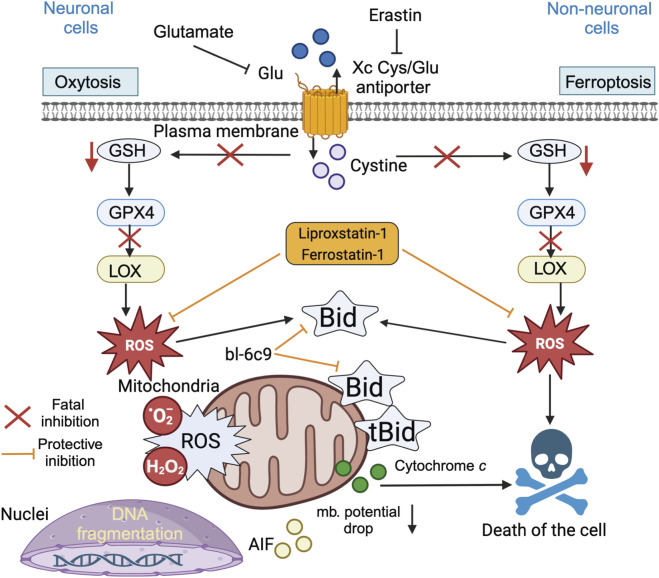
BID links ferroptosis to neuronal oxytosis. Glutamate and erastin inhibit the Xc (glutamate) antiporter in oxytosis and ferroptosis, respectively. Blockade of cellular cystine import via the Xc transporter results in lower GSH levels and decreased Gpx4 activity, leading to the activation of 12/15 lipoxygenase (LOX), which significantly generates reactive oxygen species (ROS). In erastin-induced ferroptosis, cell death is induced by oxidative stress and is independent of mitochondrial death. In neuronal cells, the ROS-induced transactivation of BID to mitochondria links both the oxytosis and ferroptosis pathways and causes the generation of mitochondrial ROS (superoxide anions and hydroperoxides), which are associated with irreversible morphological and functional damage, such as the loss of the mitochondrial membrane potential, a decrease in ATP levels, and the release of cytochrome *c* and apoptosis-inducing factor (AIF). The BID inhibitor BI-6c9 (which belongs to the BCL-2 protein family) and the ferroptosis inhibitors ferrostatin-1 and liproxstatin-1 are able to block these lethal pathways upstream of mitochondrial damage. BI-6c9 directly inhibits BID and its deleterious effects at the mitochondrial level, whereas ferrostatin-1 acts upstream of BID to prevent ROS generation via 12/15 LOX (Gan, 2023).

Indeed, the molecular mechanisms underlying oxytosis are remarkably similar, if not identical, to the cell death process termed ferroptosis several years later, when the involvement of iron-dependent lipid peroxidation (Latunde-Dada, 2017) was first described, and both pathways are currently thought to involve one and the same process. Oxytosis/ferroptosis has emerged as a potential key factor in the progression of neurodegenerative diseases, linking various mechanisms such as oxidative stress, mitochondrial dysfunction, and immune dysregulation, and it likely plays an important role in these interconnected pathways, making it a promising target for therapeutic innovation. Therefore, it is more likely that oxytosis and ferroptosis should be regarded as two names for the same cell death pathway (Lewerenz et al., 2018). Oxytosis is a paradigm of oxidative cell death that has been established in neuronal cells (Tan et al., 2001) (Figure 10). Oxytosis can be induced in neuronal cells via glutamate-mediated inhibition of the cystine-glutamate antiporter (Xc-). Such depletion of the intracellular glutathione pool decreases GSH levels (Tan et al., 2001) and impairs the redox defense of neuronal cells, resulting in increased ROS levels that can trigger caspase-independent cell death 6, 67]. In particular, GSH depletion promotes the inhibition of glutathione peroxidase-4 (Gpx4) and the enhancement of 12/15-lipoxygenase (LOX) activity, both of which are key steps upstream of mitochondrial dysfunction (Grohm et al., 2010; Tobaben JG et al., 2011; Dixon et al., 2012; Dixon, 2017). In neuronal oxytosis paradigms, mitochondrial impairment is mediated by mitochondrial transactivation of the proapoptotic protein BID (Dixon, 2017; Linkermann et al., 2014; Friedmann Angeli et al., 2014) (Figure 9). Upon translocation to the mitochondria, BID mediates the loss of mitochondrial integrity and function and the deleterious translocation of mitochondrial AIF to the nucleus (Landshamer et al., 2008; Hu et al., 2017). Ferroptosis has been described as an iron-dependent form of oxidative cell death in cancer cells and in neurons (Murphy, 2018; Shen et al., 2018; Kenny et al., 2019). Notably, ferroptotic cell death has also been observed in kidney cells (Muller et al., 2017; Li et al., 2021; Sato et al., 2018). Ferroptosis involves the generation of ROS via iron-dependent enzymatic reactions mediated by lipoxygenases, xanthine oxidases, NADPH oxidase or catalase. In addition to iron-chelating compounds, ferroptosis can be blocked by ferrostatin-1, a potent inhibitor that mediates protective effects against erastin-induced ferroptosis by inhibiting lipid peroxidation (Latunde-Dada, 2017; Conrad et al., 2018). Mechanisms of ferroptosis have also been identified in neurons, death signaling pathways induced by cerebral ischemia (She et al., 2020), glutamate-induced neurotoxicity in organotypic hippocampal slice cultures (Zou and Crews, 2005), and (Song et al., 2021). Like glutamate-induced oxytoxicity, erastin activates ferroptosis by inhibiting the Xc^−^ transporter. However, in contrast to oxytosis, ferroptosis in cancer cells is not associated with mitochondrial damage (Ding et al., 2025). In fibroblasts and kidney cells, ferroptosis is accompanied by prototypical changes in mitochondrial morphology, including reduced organelle size, the disappearance of mitochondrial cristae, increased mitochondrial membrane density, and the rupture of the mitochondrial outer membrane (Liu et al., 2023).

The emerging hypothesis is that oxytosis and ferroptosis may converge in intrinsic death signaling at the level of mitochondrial dysfunction. The ferroptotic and oxytotic pathways are clearly linked via cytoplasmic BID (Figure 10), its subsequent delocalization to the MOM surface, and classical tBID/BAX mitochondrial damage.

## 5 Conclusion

Although the above cell death modalities are defined by their respective causes and distinct morphological changes, the signaling molecules that activate cell death pathways are often interconnected owing to the pleiotropic nature of these molecules. Here, the example of BID allows us to introduce protein‒lipid interactions. Typically, apoptotic cell death, with mitochondria in a cardinal position, is important because the release of cytochrome *c* from mitochondria activates apoptosis. The resulting breakdown of the mitochondrial electron transport chain generates ROS, which may also contribute to ferroptosis.

Here, we investigated to what extent the cardinal feature of classical apoptosis in type II cells (Danial and Korsmeyer, 2004; Scaffidi et al., 1998) is important and can be partially shared by different newly defined cell death pathways. Therefore, triggering the DISC leads to the activation of caspase-8 (Peter and Krammer, 2003; Scaffidi et al., 1999). Activated caspase-8 cleaves caspase-3, leading to the induction of cell death in type I cells. In type II cells, however, this is blocked by the XIAP. However, active caspase-8 also cleaves BID and induces MOMP, which in turn leads to the release of proapoptotic factors, including cytochrome *c* and Smac/DIABLO, from the mitochondrial intermembrane space. The release of cytochrome *c* triggers the formation of the “apoptosome” and the activation of caspase-9, whereas the release of Smac/DIABLO leads to the neutralization of XIAP, allowing the full maturation and activation of the effector caspase-3, -7 and -9 and consequently the induction of apoptosis (Figure 1).

We then focused our attention on the relationships among BID, caspase-8 and cardiolipin accessible to the MOM. The use of multiple diverse methodological approaches, including biophysics and bioenergetics combined with the usual methods of cell biology, has led us to fully characterize the interactions between these third components [e.g., a BH-3-only protein, caspase-8 (an enzyme) and cardiolipin (a lipid specific to mitochondria)]. The specific interactions involved were discussed, and the formation of an activation platform (Gonzalvez et al., 2005a), the so-called “mitosome,” was clearly confirmed (Figures 3, 6). The terminal activation of caspase-8 by cardiolipin has also been demonstrated, providing the basis for a very advanced and original concept for caspase-8 (Gonzalvez et al., 2008), which only reaches full completion at the membrane. We also present some biophysical studies that push forward the current models of BH3-only protein function in the mitochondria following the activation of BID to give rise to tBID (Jalmar et al., 2010; Jalmar et al., 2013). The interaction of tBID with cardiolipin and its consequences are depicted in terms of biophysics. In terms of bioenergetics, mitochondrial bioenergetic homeostasis is destabilized by tBID (Figure 6) (Gonzalvez et al., 2005a; Gonzalvez et al., 2005b) after its interaction with the MOM cardiolipin and the oxidized CLOOH resulting from the inhibition of the electron transport chain.

In fact, there are multiple examples of the interplay between different cell death pathways. In addition to the example presented in this article concerning the platform formed by cardiolipin, caspase-8, and BID, many other intricate situations could be illustrated.

One interesting example is the activation of apoptosis and necroptosis by RIPK in a kinase-dependent manner. In this process, RIPK acts as an adaptor protein that mediates apoptosis initiated by a conformational change in RIPK3. Treatment with common chemotherapeutic agents induces caspase-9-mediated apoptosis and activates GSDME, which activates pyroptosis. Additionally, the combination of TNF-α and IFN-β or PAMPs can promote a mixed form of cell death called “PANoptosis” (a type of inflammatory lytic death triggered by innate immune sensors and mediated by caspases and RIPKs), which simultaneously activates pyroptosis, apoptosis, and necroptosis.

This phenomenon could be completed by IFNβ, since IFNβ is involved in promoting ferroptosis by inhibiting the expression of SCL7A11 and SLC3A2, which constitute part of an anti-ferroptosis system in the Xc-glutathione GPX4 axis. In addition, a membrane-trafficking system endosomal sorting complex required for transport-III (ESCRT-III) functions in membrane remodeling and scission and regulates multiple cell death modalities by facilitating the shedding and repair of the mixed lineage kinase domain like pseudokinase MLKL, gasmerdine-D (GSDMD), and peroxidized lipid-damaged plasma membranes and delaying cell death kinetics. Initially, the concept of regulated cell death was limited to apoptosis and centered around caspase-8 activation and BID (BH3-only protein). The first set of genes involved in regulated cell death was related to apoptosis. However, this view has evolved rapidly, as researchers have described the molecular mechanisms of other forms of regulated cell death, including necroptosis, pyroptosis, and ferroptosis, which appear to be very complex. More precise knowledge of cell death mechanisms allows the identification of specific biomarkers for each form of cell death in tissues. This is central to exploring the pathological roles of these different forms of cell death**.**

An important and compelling question is how many other forms of regulated cell death with distinct intracellular pathways remain to be discovered. Interestingly, these pathways, or crosstalk, are often not detectable. They are only activated when another pathway is inhibited. For example, deletion of caspase-8 or mutation of its auto-processing sites activates RIP3/MLKL-dependent necroptosis, a pathway not otherwise known. Catalytically dead caspase-8 also activates necroptosis and pyroptosis.

The mitochondrial cristae organizing system (MICOS) is increasingly recognized as a fundamental determinant of mitochondrial membrane architecture and physiology. Its intimate crosstalk with many other mitochondrial protein machineries identifies the MICOS as a central hub in an intertwined network that ensures the functionality and integration of mitochondria in many cell death processes that remain to be widely investigated.

Knowledge and tools developed from studying the biochemical pathways of these four well-defined types of regulated cell death will undoubtedly lay the foundation for identifying other types of regulated cell death that are distinct from apoptosis, necroptosis, pyroptosis, and ferroptosis.

## Author’s note

The title is inspired voluntarily from the review article by Kantari Ch. Walczak H. recognized for the first time the importance of a platform of activation located at the mitochondrial membrane.
